# Recent advances in the prevention and management of infections in children undergoing treatment for cancer

**DOI:** 10.12688/f1000research.19337.1

**Published:** 2019-11-12

**Authors:** Bob Phillips

**Affiliations:** 1 Centre for Reviews and Dissemination, University of York, York, Yorkshire, YO10 5DD, UK; 2 Leeds Children’s Hospital, Leeds General Infirmary, Leeds, Yorkshire, LS1 9EX, UK

**Keywords:** febrile neutropenia, neutropenia, supportive care, pediatric oncology

## Abstract

A major consequence of the intensive multi-modal chemotherapy commonly used to treat malignancies in childhood is life-threatening infection, frequently during periods of profound neutropenia. Recent advances have been made in all areas of management, from trying to prevent infection to getting patients off antimicrobials and home again in the shortest, safest way. Potential avenues of further research are outlined for readers to be aware of in the next few years.

## Background

The knotty problem of dealing with children undergoing immunosuppressive treatment for cancer to prevent and treat, but not overtreat, potential life-threatening infection continues to intrigue and enrage clinical academics working in this field. The challenges lie in reducing the chance of an infection developing and leaping upon infections quickly to maximize the likely outcomes but discontinuing antibiotic treatments and hospitalization to reduce the adverse psychological, social, and medical effects of being an in-patient. A growing body of primary studies and systematic reviews is assisting with these areas and identifying where further studies are required.

## Preventing infection

Prevention of infection has been addressed by reducing the chance of acquiring an infection and by using prophylactic antibacterial therapy. Systematic reviews of the use of specialized “low-bacterial diets” to decrease the risk of bacterial translocation across the gut wall have demonstrated little benefit for this approach ^1^, which is confirmed in a specific comparative study in children ^2^. These diets are harder to adhere to for families, contain far less joy, and are convincingly unhelpful. Other traditional, but unevaluated, practices such as “protective” isolation or hospitalization and the wearing of facemasks or avoidance of companion animal contact deserve the same sort of scrutiny as “low bacterial diets” in the future.

The concept of infections being affected by gut bacteria is not without merit, though. Analysis of the gut microbiome in children being treated for acute lymphoblastic leukemia (ALL), the commonest single childhood malignancy, shows association between particular patterns of colonization and infection ^3^. Evidence synthesis of trials of probiotics shows a reduction in fever episodes ^4^, though there are few trials in children and a hint of “small study effects”, which may have produced overoptimistic results. The same review demonstrated a remarkably low level of probiotic-associated sepsis, and all infections were easily and effectively treated with antimicrobials. This element of iatrogenic harm from probiotics appears to be based more on fear than solid evidence.

Rather than introducing controlled bacteria, an opposing approach has been used in children at highest risk of invasive infection: the use of prophylactic antibiotics. A large systematic review of trials in all ages demonstrated a reduction in fever episodes, proven infections, and death ^5^. There are very few studies in children, though; the only large, modern-era study did show benefit for levofloxacin in a group at particularly high risk of infection (ALL and relapsed, heavily immunosuppressed ALL patients) ^6^. Whether this is the right way to use antibiotics remains hotly contested, as the use of prophylaxis raised the proportion of isolates resistant to the antibacterials. Though this wasn’t a clinical problem in the time frame of the trial, commentators, particularly those who are in countries with high rates of antimicrobial resistance, raise valid questions around this approach ^7^.

Despite the widespread use of granulocyte colony stimulating agents, very few recent studies have been undertaken to examine them. Systematic reviews from a decade ago found little evidence to support their prophylactic use in children ^8^. Specific regimens have found benefit broadly in terms of side effects when treating neuroblastoma induction chemotherapy ^9^, but broader studies have yet to provide convincing evidence of benefit with modern, restrictive, antibiotic stewardship approaches.

## Antibacterial approaches

Antibiotic delivery to patients who present with signs of potential infection, which in this group is classically fever in the presence of neutropenia, is widely accepted to be a universal “good thing” ^10^. There remains some reasonable debate as to whether the “one-hour window” for antibiotic delivery is useful: a very recent systematic review of well-conducted studies showed an association between longer duration to treatment and increased risk of adverse outcomes but could not draw a line in the sand at 60 minutes ^11^. While there have been some approaches to define a group in whom no antibiotics are necessary, this hasn’t been shown to be practical. The focus has moved to defining how narrow a spectrum of antibacterial can be safely used ^12^ and ways of discontinuing treatment quickly. Research has confirmed patients and their families want to be treated as safely as possible but get out of hospital as soon as they can to improve their life experience ^13, 14^.

The discontinuation drive forks at those attempts to define, at presentation of the feverish episode, a way of clarifying who doesn’t need intravenous antibiotics and admission; and attempts to describe who can have antibiotics stopped. Up-front clinical prediction models have been widely generated but infrequently tested and validated ^15^. When they have, they have shown only moderate value and require refinement ^16, 17^. Practically, implementing a method of risk stratifying and treating using such a rule has been shown to be relatively safe, both across all ages ^18^ and specifically in children ^19^. The approaches tend to use a combination of factors related to the malignant diagnosis of the child, their social situation, and episode-related elements. In this way, those treated as out-patients would not have received conditioning chemotherapy for a hematopoietic stem cell transplant, live hours from medical attention, or have arrived in hospital with septic shock and a need for inotropic support.

Stopping rules have been investigated relatively little, in comparison with other aspects of management, and a traditional approach of continuing antibacterials until the fever has settled, often for 48 hours, and the bone marrow shows signs of recovery is still common practice ^10^. Challenges to this have been made, questioning the need for count recovery in clinically well children ^20, 21^ or stopping treatments earlier when a definitive viral infection without co-existing severe bacterial infection has been documented ^22^. Such “enhanced stopping” approaches may be more effective than a risk stratification system, which at best affects 30% of patients. Other approaches which harness dynamic changes in easily measurable serum biomarkers, such as procalcitonin, are also set for clinical trials in this group ^13^.

## Persistent fevers and fungus

As well as a group of children in whom a confirmed bacterial infection requires ongoing antibacterial therapy, there is a group with persistent fever in whom no clear cause is immediately identified. The worry in these heavily immunocompromised patients is of invasive fungal disease (IFD). A systematic review pulled all of the available data together to try to distinguish those who would be at higher and lower risk in order to rationally target the use of empirical antifungal drugs, medications which have high costs and tricky side effects ^23^. The review showed a disappointing absence of well-conducted studies and failed to describe really clear groups which had very low or very high risks. Relapsed and intensively treated leukemias, those where neutropenia was expected to be profound and long-lasting, those undergoing treatment with high doses of corticosteroids, and those with graft-versus-host disease were confirmed to be the greatest worry. With difficulty in determining risk groups precisely once again facing clinicians, attention has been brought to bear on the use of empiric antifungals, where the treatment is given to everyone at moderate–high risk, or the use of a pre-emptive approach, in which only in the presence of early imaging or biomarker signs of invasive fungal infection are the antifungal agents commenced. Both strategies have been used, and a randomized trial in children seems to suggest that they have equivalent safety, with a reduced use of medication in the pre-emptive arm ^24^. The key element in this pre-emptive approach is “screening” for hints of infection and looking to rule out an emerging IFD. There is a growing body of tests based on bodily fluids, blood, and alveolar or cerebrospinal fluid, which could make this approach even more effective. Combining markers of the most common invasive species, and broad pan-fungal PCR detection approaches, could be this improvement ^25^. Even with this suggestion of future improvement, there remains some concern; the results of a single, relatively small trial should not determine treatment in a condition with a very high fatality rate and, as with many areas of medicine, it may remain debated hotly until further studies are undertaken.

## Conclusion

Infection in the child undergoing cancer treatment remains a frequent, occasionally fatal, and better manageable complication of therapy. The current standard of care is that children with cancer who have fever and neutropenia are evaluated promptly and antibiotics are initiated. This may be in the hospital or at home, depending on the underlying diagnosis of the child, their social situation, and episode-related elements. In the future, we may be modifying the microbiome with probiotics, giving judicious prophylaxis to patients at highest risk of infection, sliding a small proportion home from the assessment cubicles, and quickly striking through the prescriptions of those who don’t have signs of serious infections, perhaps guided by inflammatory biomarkers. Persistent fevers will remain troubling to us, but more research will be undertaken to try to elucidate who is developing invasive fungal infections and how we can effectively treat them.

## Abbreviations

ALL, acute lymphoblastic leukemia; IFD, invasive fungal disease
